# A Systematic Review and Meta-Analysis of Anterolateral Thigh Flap Outcomes in High-Risk Diabetic Foot Reconstruction

**DOI:** 10.3390/jcm14238481

**Published:** 2025-11-29

**Authors:** Abdalah Abu-Baker, Andrada-Elena Ţigăran, Teodora Timofan, Daniela-Elena Ion, Daniela-Elena Gheoca-Mutu, Adelaida Avino, Adrian Daniel Tulin, Laura Raducu, Cristian-Radu Jecan

**Affiliations:** 1 Doctoral School, “Carol Davila” University of Medicine and Pharmacy, 010221 Bucharest, Romania; abdalah.abu-baker@drd.umfcd.ro (A.A.-B.); andrada-elena.tigaran@drd.umfcd.ro (A.-E.Ţ.); 2 Discipline of Plastic Surgery, “Carol Davila” University of Medicine and Pharmacy, 010221 Bucharest, Romania; teodora.peligrad@rez.umfcd.ro (T.T.); daniela-elena.ion@rez.umfcd.ro (D.-E.I.); adelaida.avino@umfcd.ro (A.A.); cristian.jecan@umfcd.ro (C.-R.J.); 3 Department of Plastic Surgery, “Prof. Dr. Agrippa Ionescu” Emergency Clinical Hospital, 011356 Bucharest, Romania; daniela-elena.mutu@umfcd.ro; 4 Discipline of Anatomy, “Carol Davila” University of Medicine and Pharmacy, 010221 Bucharest, Romania; 5 Department of General Surgery, “Prof. Dr. Agrippa Ionescu” Emergency Clinical Hospital, 011356 Bucharest, Romania

**Keywords:** anterolateral thigh flap, diabetic foot ulcer, diabetic foot reconstruction, free tissue transfer, systematic review, meta-analysis, flap survival, flap outcomes

## Abstract

**Background**: Complex diabetic foot ulcers (DFUs) are a leading cause of morbidity and lower-limb amputation, and their management is profoundly challenging. Microvascular free tissue transfer is a primary limb salvage strategy, with the anterolateral thigh (ALT) free-flap recognized as a workhorse reconstructive solution. However, a quantitative summary of its performance specifically within this high-risk patient population is lacking. **Methods**: A systematic review and single-arm meta-analysis was conducted in accordance with PRISMA (Preferred Reporting Items for Systematic Reviews and Meta-Analyses) guidelines. Five electronic databases (PubMed/MEDLINE, Embase, Scopus, Cochrane, and Web of Science Core Collection) were searched up to 9 September 2025 to identify studies reporting on outcomes of ALT free-flaps for diabetic foot reconstruction. The risk of bias was assessed using the Methodological Index for Non-Randomized Studies (MINORS) criteria. Primary outcomes were set as the complete and partial flap necrosis rate. Secondary outcomes included functional recovery status and complication rates. All data were synthesized using a random-effects model. **Results**: Six retrospective cohort studies met the inclusion criteria, including a total of 162 patients. The pooled rate of total flap failure was 5.2% (95% CI: 2.5–10.6%), a finding that was highly consistent across all studies (I^2^ = 0%). The pooled incidence of partial flap necrosis was 13.0% (95% CI: 6.3–25.1%), resulting in an overall weighted flap success rate of 81.8%. Notably, the pooled rate of return to ambulation was 95.2% (95% CI: 88.5–98.1%), which also demonstrated no statistical heterogeneity (I^2^ = 0%). **Conclusions**: The anterolateral thigh free-flap appears to be a robust and highly reliable strategy for diabetic foot reconstruction, associated with low failure rates, minimal long-term complications, and excellent functional recovery. However, the current evidence is limited to a small number of poor-to-moderate-quality retrospective studies. High-quality, prospective, and comparative multicenter trials are necessary to confirm these findings and establish the ALT flap’s effectiveness in high-risk cohorts.

## 1. Introduction

Diabetes mellitus represents a growing global health challenge, placing considerable strain on communities and healthcare systems. The global rise in disease prevalence is well-documented, with an estimated 529 million individuals worldwide living with diabetes, in whom type 2 diabetes accounts for around 90–95% of all cases [1,2]. This condition significantly contributes to global mortality and disability, ranking among the top 10 causes of death worldwide [3]. Moreover, the economic ramifications are considerable, as worldwide healthcare expenses associated with diabetes have a notable rising trajectory [4]. Notably, this disease burden is disproportionately shifting, with the greatest impact now trending toward low- and middle-income countries [4,5].

Diabetic foot ulcers (DFUs) represent a significant source of morbidity and mortality in the diabetic population [6]. Diabetic foot ulcers are the principal factor in over 85% of diabetes-related lower limb amputations, leading to severe consequences, including 5-year mortality rates over 70% and recurrence rates reaching 65% [7,8,9]. Infection complicates these chronic wounds in 50–60% of patients, resulting in longer hospital stays and an increased risk of sepsis and osteomyelitis [10]. The etiology of DFUs is driven by neuropathy, vascular insufficiency, and impaired wound healing. These pathologic pathways are exacerbated by risk factors such as poor glycemic control, obesity, hypertension, peripheral vascular disease, and foot deformities [11,12].

The management of complicated diabetic foot ulcers is significantly challenging due to a combination of biological and clinical variables. Healing is frequently hindered by persistent inflammation, marked by dysregulated macrophage function and excessive breakdown by matrix metalloproteinases (MMPs), particularly MMP-9, which inhibits tissue repair [13,14,15]. This non-healing wound environment is highly susceptible to infection, frequently complicated by the formation of antibiotic-resistant bacterial biofilms that undermine both host defenses and standard treatments [16,17]. Concurrently, underlying comorbidities such as peripheral artery disease and mechanical stress from neuropathy necessitate specialized, often conflicting, interventions [18,19]. Even when wound closure is achieved, patients face high recurrence rates and significant long-term mortality [20]. These clinical challenges are frequently aggravated by inconsistent care delivery and the irregular use of evidence-based protocols, thus resulting in significant obstacles to achieving optimal results [21,22].

Microvascular free tissue transfer (free-flaps) is a primary limb salvage strategy for complex diabetic foot ulcers involving extensive tissue loss or infection [23]. This approach demonstrates high limb salvage (85–95%) and flap survival (92–98%) rates in appropriately selected patients [24,25]. A multidisciplinary strategy centered on preoperative optimization, including infection control, comorbidity management, and vascular assessment, is crucial to the success of the procedure [26,27]. For ischemic limbs, free-flap reconstruction is frequently combined with surgical or endovascular revascularization [28]. While both muscle and fasciocutaneous flaps are effective, careful patient selection is essential to reduce complications [29]. Severe peripheral vascular disease and poor glycemic control have been shown to be associated with higher rates of subsequent amputation [30].

The anterolateral thigh free-flap is a crucial reconstructive technique for complex lower extremity defects [31]. It consistently demonstrates high survival rates and provides robust, shear-resistant coverage critical for weight-bearing [32]. It is particularly indicated for extensive or distal defects where local tissue is insufficient. Its utility is derived from its favorable characteristics, which include a large, pliable skin paddle, a long vascular pedicle, and minimal donor-site morbidity [33]. This flap’s versatility is further enhanced by its potential to be harvested with a muscle component, such as the vastus lateralis muscle, to manage dead space or infection, or as a sensate flap, in order to reconstruct plantar sensibility [34].

This systematic review and single-arm meta-analysis aims to synthesize the available evidence on the outcomes of anterolateral thigh free-flaps for the reconstruction of diabetic foot wounds. The ALT flap is a well-established workhorse in reconstructive surgery; nevertheless, a quantitative summary of its efficacy specifically within this high-risk patient population is not available.

The primary and secondary objectives of this study were established to comprehensively address this knowledge gap:

Primary Objective:

- To determine the pooled flap survival rate, categorized as complete survival, partial necrosis, or total failure.

Secondary Objectives:

- To synthesize data on wound-healing parameters.
- To determine the pooled incidence of postoperative complications.
- To evaluate intraoperative variability and technical flap characteristics.
- To assess the long-term outcomes, such as ulcer recurrence, mortality, and return to ambulation.
- To report available data on patient-reported quality of life.

## 2. Materials and Methods

The authors have prepared this manuscript ensuring that it strictly follows the standards of the PRISMA (Preferred Reporting Items for Systematic Reviews and Meta-Analyses) guidelines protocol for systematic reviews and meta-analytic studies (Supplementary Material). However, this study protocol was not registered, as PROSPERO does not yet provide support for single-arm proportional meta-analyses.

### 2.1. Study Eligibility

A predefined set of inclusion and exclusion criteria was used to guide the selection of studies for this review, ensuring a focused and relevant synthesis of the evidence.

Inclusion Criteria:

- Adult patients (aged ≥ 18 years) with a diagnosis of diabetes mellitus presenting with foot or ankle defects requiring surgical reconstruction.
- Evaluated the use of the anterolateral thigh free flap for reconstruction. Eligible studies included those that either:
  ○ Directly compared the ALT free flap to other free tissue transfers.
  ○ Reported on a single-arm case series of ALT flaps with at least five patients.
- Eligible study designs included randomized controlled trials (RCTs), prospective and retrospective cohort studies, case–control studies, and comparative or single-arm case series (as defined above).
- Reported on at least one of the predefined primary outcomes.
- Published in the English language (or had a full English translation available), with no restrictions placed on the date of publication.

Exclusion Criteria:

- Focused on non-diabetic wound etiologies (e.g., acute trauma, malignancy) where data for the diabetic subgroup could not be isolated.
- Evaluated reconstruction with non-microvascular techniques, such as local pedicled flaps or skin grafts, microsurgical reconstruction without using the ALT flap.
- Were case reports with fewer than five patients, technical notes lacking clinical outcome data, narrative reviews, editorials, letters, or conference abstracts without a corresponding full-text publication.
- Reported on reconstructions for anatomical sites other than the lower limb.

### 2.2. Search Strategy

A comprehensive and systematic literature search was conducted to identify all relevant studies. The search was performed across five major electronic databases: PubMed/MEDLINE, Embase, Scopus, the Cochrane Library, and the Web of Science Core Collection.

The search was conducted from the inception of each database up to 9th of September 2025, the date when the search occurred. A systematic search strategy was developed for PubMed/MEDLINE and subsequently adapted for the syntax and subject headings of the other databases. The strategy combined Medical Subject Headings (MeSH) with keywords related to three core concepts: the diabetic foot disease, microvascular free tissue transfer, and the anterolateral thigh flap. No date or language restrictions were initially applied to the search, though only English-language articles were included in the final analysis as per the eligibility criteria.

The full search strategy used for PubMed/MEDLINE is detailed below:

(“Diabetic Foot”[Mesh] OR diabetic foot[tiab] OR diabetic feet[tiab] OR foot ulcer\*[tiab] OR diabetic ulcer\*[tiab] OR “Charcot foot”[tiab]) AND (“Free Tissue Flaps”[Mesh] OR free flap\*[tiab] OR free tissue transfer\*[tiab] OR microsurg\*[tiab]) AND (“Anterolateral Thigh”[tiab] OR “anterolateral thigh”[tiab] OR ALT[tiab] OR ALT flap\*[tiab]) AND (foot[tiab] OR feet[tiab] OR pedal[tiab] OR plantar[tiab] OR ankle[tiab])

The adapted full search strategy used for the other databases is presented in Appendix A.

### 2.3. Study Selection

The study selection was conducted systematically and transparently to ensure an unbiased and reproducible process.

Initially, all records identified from the database searches were exported into a reference management software (Rayyan, version 1.4.3), where duplicate records were identified and removed [35]. Subsequently, the selection process was performed in two distinct stages by two independent reviewers (A.A.-B. and A.-E.Ț.).

In the first stage, the reviewers independently screened the titles and abstracts of all unique records against the predefined eligibility criteria. Records that were clearly irrelevant were excluded. In the second stage, the full texts of all potentially relevant articles were retrieved and assessed independently by the same two reviewers. During this phase, the inclusion and exclusion criteria were applied rigorously to determine final eligibility.

Any disagreements between the two reviewers that arose during the full-text screening stage were resolved through discussion and consensus. If a consensus could not be reached, a third, senior reviewer (L.R.) was consulted to make the final decision.

The entire study selection process was documented in detail and is summarized in a PRISMA 2020 flow diagram, which illustrates the flow of information through the different phases of the review, including the number of records identified, included, and excluded [36].

### 2.4. Data Extraction

Two reviewers (A.A.-B. and A.-E.Ț.) independently extracted data from each included study using a standardized data collection form, data pertaining to the primary and secondary objectives of the study. Any discrepancies in extracted data between the two reviewers were resolved through discussion and consensus, with a third reviewer (L.R.) available for arbitration if necessary.

The following data items were extracted from each study, where available:

- Study Characteristics: First author, year of publication, country of origin, study design, and total sample size.
- Population Demographics: Mean patient age, mean Glycated Hemoglobin (HbA1c), prevalence of key comorbidities (chronic heart disease, chronic kidney disease, peripheral artery disease, smoker status, osteomyelitis), and anatomical location of the defect
- Surgical Details: Mean flap size, pedicle length, number of perforators detected, type of microvascular anastomosis, and the recipient vessels used.
- Postoperative Outcomes: Rates of complete and partial flap necrosis, incidence of complications (including infection and vascular compromise), surgical revision rates, and the mean length of hospital stay.
- Long-Term Outcomes: Mean duration of follow-up, rates of ulcer recurrence and mortality, wound healing metrics (including the percentage of healed wounds and the mean time to complete healing), functional outcomes (such as the number of patients who achieved ambulation), and any patient-reported quality of life measures.

### 2.5. Risk of Bias Assessment

The methodological quality of the selected studies was rigorously evaluated to determine the risk of bias. Two reviewers (A.A.-B. and A.-E.Ț.) independently evaluated each study, and any discrepancies in their assessments were resolved through discussion and consensus.

Given that the included studies were predominantly non-randomized, single-arm case series, the Methodological Index for Non-Randomized Studies (MINORS) criteria were used. The MINORS tool is specifically designed to assess the quality of non-comparative and comparative surgical studies, focusing on key aspects of internal validity like clear statement of aims, inclusion of consecutive patients, prospective data collection, and unbiased assessment of outcomes [37].

### 2.6. Statistical Analysis

A single-arm meta-analysis was performed to synthesize the primary and secondary outcomes reported as proportions, such as flap survival and limb salvage rates, or as means, such as mean age or mean HbA1c levels. All statistical analyses were conducted using Jamovi (Version 2.3) with the MAJOR meta-analysis module [38].

Due to the nature of proportional data, which often does not follow a normal distribution, a logit transformation was applied to the raw proportions from each study. The transformed data were then pooled using a random-effects model. This model was chosen a priori as it accounts for both the within-study sampling error and the between-study variance. The pooled means for continuous variables were determined using the mean, standard deviation, and sample size of each parameter. Statistical heterogeneity among the studies was quantified using the I^2^ statistic. The significance of this heterogeneity was assessed using the Cochran’s Q (Chi-squared) test.

The results of the meta-analysis for each outcome were visually represented using a forest plot, using a 95% confidence interval. Publication bias was assessed both visually using a funnel plot and statistically using Egger’s linear regression test.

Subgroup analysis was planned for each instance of significant statistical heterogeneity (I^2^ > 50%), using characteristics such as occurrence of osteomyelitis or peripheral arterial disease. The feasibility of this analysis was dependent on the number of included studies and consistent reporting of these variables.

A sensitivity analysis was performed to evaluate the robustness of the pooled estimates of primary outcomes, using a fixed-effect model. Moreover, we excluded studies identified as having a high risk of bias according to the MINORS criteria and recalculated the pooled proportions to assess whether the results differed significantly.

The overall certainty in the body of evidence was assessed using the GRADE (Grading of Recommendations Assessment, Development and Evaluation) framework [39].

## 3. Results

### 3.1. Study Identification

The initial database search identified 253 records (PubMed/MEDLINE: 16, Embase: 26, Scopus: 49, Cochrane: 137, Web of Science: 25). After 66 duplicate records were removed, 187 unique articles remained for screening.

During the title and abstract screening, 167 records were excluded as they did not meet the eligibility criteria. The remaining 20 reports were sought for full-text retrieval, of which one could not be retrieved. This left 19 full-text articles to be assessed for eligibility.

Following the detailed full-text review, 13 studies were excluded for the following reasons: article not in English (*n* = 4), data for diabetic foot cases were not analyzed separately from other etiologies (*n* = 4), or outcomes for ALT flaps were not reported separately from other reconstruction types (*n* = 5).

**Figure 1:**
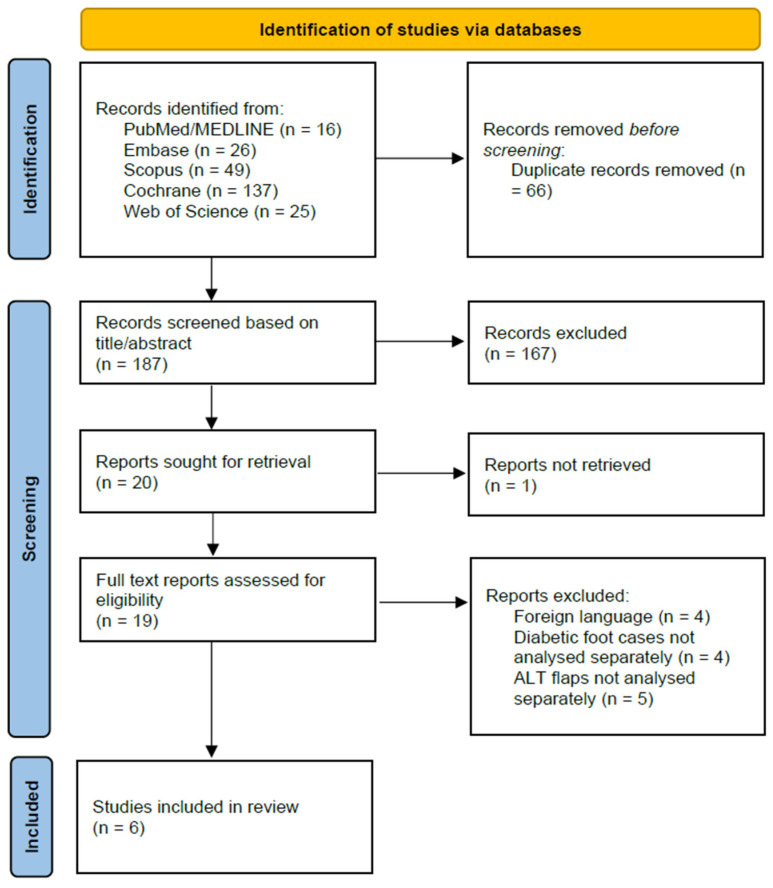
PRISMA flow diagram.

Ultimately, this process resulted in six studies that met all inclusion criteria and were included in the final systematic review and meta-analysis. The complete study selection process is illustrated in the PRISMA flow diagram (Figure 1).

### 3.2. Study Characteristics

A total of six studies, published between 2005 and 2025, met the inclusion criteria. These studies collectively included 162 patients who underwent anterolateral thigh free flap reconstruction for diabetic foot defects. All six studies were retrospective cohorts conducted in single centers in Asia. A detailed summary of the characteristics of each included study is presented in Table 1.

The mean age of patients across the studies ranged from 42.8 to 62.8 years. The study populations were predominantly male. The follow-up periods varied significantly, with a mean follow-up ranging from 6 months to 48 months.

The inclusion criteria varied slightly among the studies. Hong 2005 [40] included a mixed cohort of patients with chronic osteomyelitis, 10 of whom had diabetic foot ulcers. Hong 2006 and Kim 2007 [41,42] specifically focused on patients with diabetic feet reconstructed with anterolateral thigh (ALT) free flaps. Kadam 2016 [43] included patients with trophic ulcers from various etiologies, with 13 cases being diabetic neuropathy. The two most recent studies were comparative cohorts. Yang 2024 [44] compared outcomes in a diabetic foot group (n = 40) to a non-diabetic group (n = 43), while Wen 2025 [45] compared an ALT flap group (n = 24) to a control group receiving sharp debridement and standard wound dressing (n = 24).

**Table 1:** Study characteristics.

First Author and Year of Study	Sample Size	Study Design	Inclusion Criteria	Age Mean	Outcome	Follow-Up Mean (Months)	Minors
Kim 2007 [42]	16	Unspecified	Diabetic feet reconstructed with free flaps	62.8	Complete necrosis: 6.25% Partial necrosis: 25% Necrosed area <10% of flap area in all cases of partial flap necrosis.	N/A	7
Hong 2006 [41]	71	Retrospective cohort	Diabetic patients with infected foot ulcers Reconstruction with the ALT free-flap	51.4	Complete necrosis: 1.4% Partial necrosis: 5.6% Unassisted bipedal gait: 95.7%	11	7
Hong 2005 [40]	28	Retrospective cohort	Chronic osteomyelitis of the lower extremity Reconstruction with anterolateral thigh perforator flaps.	42.8	Complete necrosis: 0% Partial necrosis: 7.1%	18.2	9
Kadam 2016 [43]	26	Retrospective cohort	Trophic ulcers of the insensate sole Microsurgical reconstruction with free-flaps	44.69	Flaps used: ALT flap (n = 18) Radial artery forearm flap (n = 4) Gracilis flap (n = 4) Complete necrosis: 0% Partial necrosis: 3.8%	48	6
Yang 2024 [44]	83	Retrospective cohort with matched controls	Inclusion Criteria for Diabetic Patients: (1) Diagnosis of Type 2 Diabetes Mellitus (2) Wagner grade ≥ 2 (3) Soft tissue defect of the foot with exposed wounds of important tissues such as bones, nerves, and tendons (4) Limb salvage patients undergoing prosthesis using flap technique Inclusion Criteria for Non-Diabetic Patients: (1) The patient’s age, defect site, defect size, and severity were similar to those of diabetic foot patients (2) Wound repair using ALT	56.5	Diabetic Foot Group: Complete necrosis: 5% Partial necrosis: 10% Non-Diabetic Foot Group: Complete necrosis: 0% Partial necrosis: 2.3% (*p* = 0.019)	27.4	16
Wen 2025 [45]	48	Retrospective cohort with matched controls	(1) Age range of 21–90 years; (2) Diagnosis of Diabetes Mellitus, either type 1 or type 2; (3) Patients exhibiting open lesions in the foot and ankle region, classified as Meggitt-Wagner grade 3 or 4.	46.1	Complete necrosis: 8.3% ALT group wound healing: 33.7 days Control group (sharp debridement and wound dressing) wound healing: 69.29 days (*p* < 0.001)	6	16

### 3.3. Risk of Bias

The methodological quality of the six included studies revealed an overall poor to moderate quality of evidence across the studies. For the four non-comparative studies, the total MINORS scores ranged from 6 to 9 out of a possible 16. The two comparative studies both scored 16 out of a possible 24. A summary of the assessment is presented in Table 2.

No studies reported prospective data collection. Furthermore, the potential for detection bias was high, as none of the studies described a blinded assessment of the primary endpoints. Finally, no study reported a calculation of the required sample size in advance.

On the other hand, all studies were found to have clearly stated aims, and the majority reported the inclusion of consecutive patients, which mitigates selection bias. For the two comparative studies (Yang 2024 and Wen 2025 [44,45]), the methodology was deemed adequate in terms of having contemporary groups, baseline equivalence between the groups, and appropriate statistical analyses.

### 3.4. Meta-Analysis of Primary Outcome

The primary outcome of flap survival was analyzed by calculating the pooled proportions for total flap failure and partial flap necrosis, with an overall flap success rate of 81.8%.

**Figure 2:**
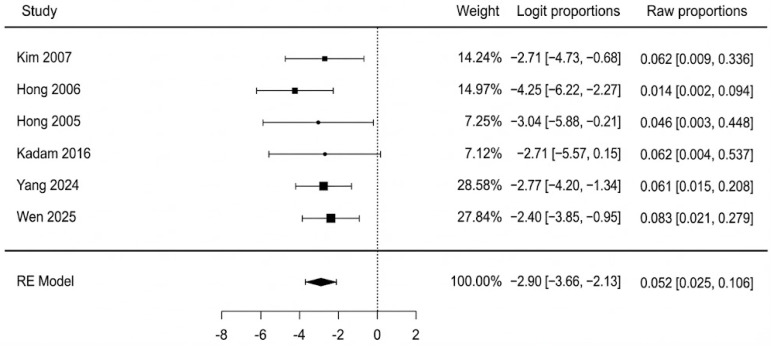
Complete flap failure forest plot [40,41,42,43,44,45].

**Figure 3:**
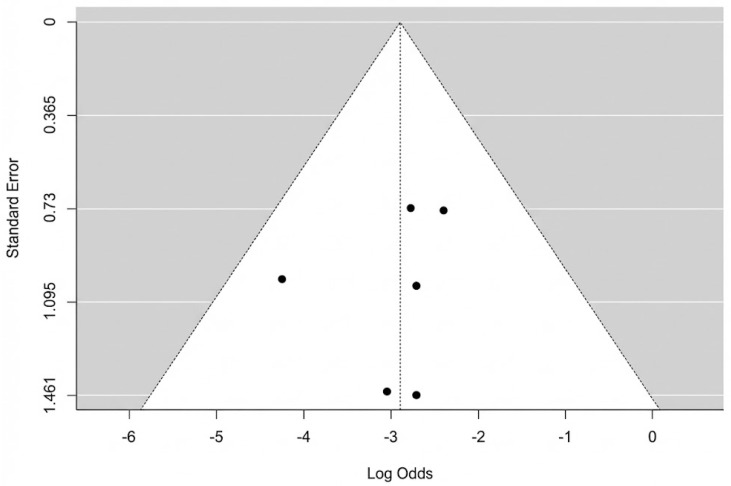
Complete flap necrosis funnel plot.

The pooled analysis of all six studies found a total flap failure rate of 5.2% (95% CI: 2.5–10.6%). The analysis showed no evidence of heterogeneity among the studies, with an I^2^ statistic of 0% (Q = 2.35, *p* = 0.799), indicating high consistency across the results. A forest plot summarizing the individual study data and the overall pooled estimate is presented in Figure 2. We re-ran the analysis using just the three moderate-quality studies (Hong 2005, Yang 2024, Wen 2025 [40,44,45]) in order to evaluate the robustness of this finding. In this sensitivity analysis, the pooled rate of complete flap failure was 6.7% (95% CI: 2.7–15.7%). This result demonstrates that, even when limited to the best available evidence, the statistical analysis remains reliable. The potential for publication bias for the outcome of complete necrosis was assessed. Visual inspection of the funnel plot revealed a symmetrical distribution of the six included studies (Figure 3). This was supported by Egger’s regression test, which was non-significant (*p* = 0.647), suggesting no strong evidence of publication bias affecting the pooled estimate.

**Figure 4:**
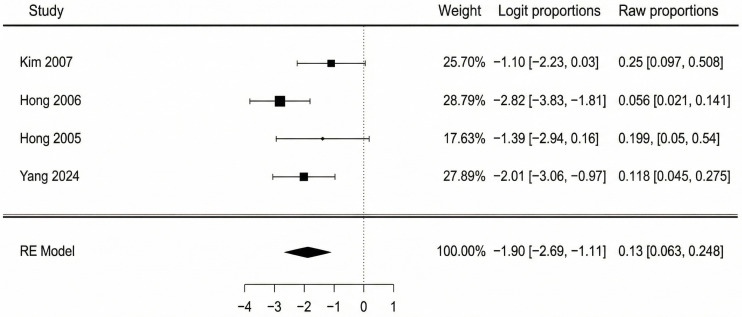
Partial flap necrosis forest plot [40,41,42,44].

Data from four studies were available for the analysis of partial flap necrosis. The pooled incidence of partial necrosis was 13.0% (95% CI: 6.3–25.1%). There was moderate heterogeneity observed among the studies (I^2^ = 46.8%, Q = 5.54, *p* = 0.136). A forest plot detailing these findings is shown in Figure 4. The potential for publication bias was also assessed. The funnel plot appeared reasonably symmetrical upon visual inspection, and this was supported by a non-significant Egger’s test (*p* = 0.333). Testing for sensitivity analysis, we performed the same assessment using a fixed-effect model, with a very similar result: 12.5% (95% CI: 7.4–20.0%). Excluding the two lower-quality studies (Kim 2007 and Hong 2006 [41,42]), the pooled analysis resulted in an incidence of 14.0% (95% CI: 6.0–28.0%) with no observed heterogeneity (I^2^ = 0%). These findings correlate closely with the primary estimate, indicating robustness to both risk-of-bias and choice of statistical model sensitivity, moreover suggesting that the heterogeneity was likely introduced by lower-quality studies. 

Combining these results, the overall weighted flap success rate was 81.8%.

### 3.5. Meta-Analysis of Secondary Outcomes

Where available, several key secondary outcomes were meta-analyzed to provide a broader clinical context for the use of ALT flaps.

**Figure 5:**
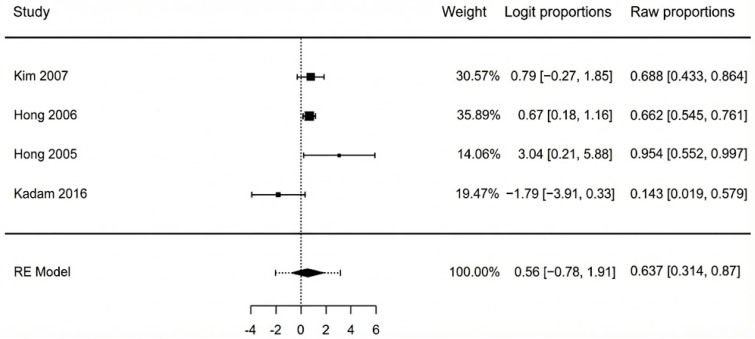
Forest plot of osteomyelitis pooled means [40,41,42,44].

Four studies reported on the prevalence of osteomyelitis in the patient cohorts requiring reconstruction. The pooled prevalence was 63.7% (95% CI: 31.4–87.0%), as shown in the forest plot of Figure 5 This result should be interpreted with caution due to high and statistically significant heterogeneity among the studies (I^2^ = 76.5%, *p* = 0.048). This indicates that the rates of osteomyelitis reported in the individual studies were substantially different from one another, making the pooled average less certain. Egger’s test was non-significant (*p* = 0.793), suggesting the variation was not due to publication bias. Subgroup analysis was not performed due to the small number of studies available for this outcome.

**Figure 6:**
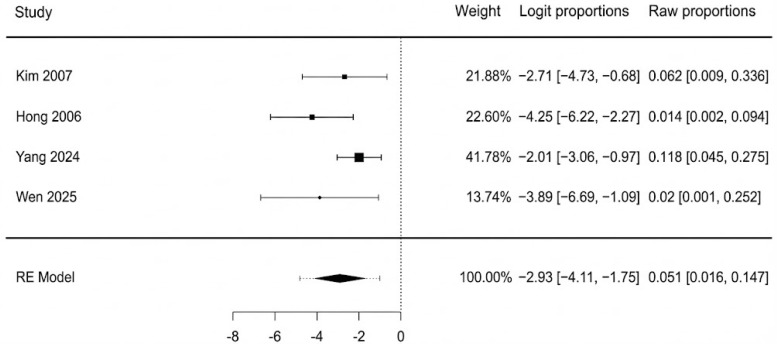
Postoperative flap infection forest plot [42,41,44,45].

The pooled incidence of postoperative flap infection, analyzed across four studies, was 5.1% (95% CI: 1.6–14.7%). The analysis showed low-to-moderate, non-significant heterogeneity (I^2^ = 40.2%, *p* = 0.195). The Egger’s test for publication bias was borderline significant (*p* = 0.070), which suggests that small studies with higher infection rates may be underrepresented, and this result should be interpreted with some caution. Pooled means are represented in Figure 6.

**Figure 7:**
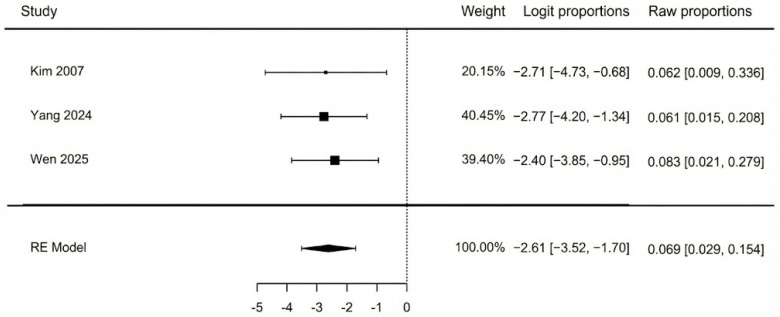
Vascular impairment [42,44,45].

The meta-analysis of three studies (Figure 7) showed that the pooled incidence of flap compromise due to vascular insufficiency was 6.9% (95% CI: 2.9–15.4%). There was no heterogeneity detected among the studies (I^2^ = 0%, *p* = 0.932), which indicates a high consistency across their findings. Additionally, a non-significant Egger’s test (*p* = 0.927) confirmed that there was no evidence of publication bias.

**Figure 8:**
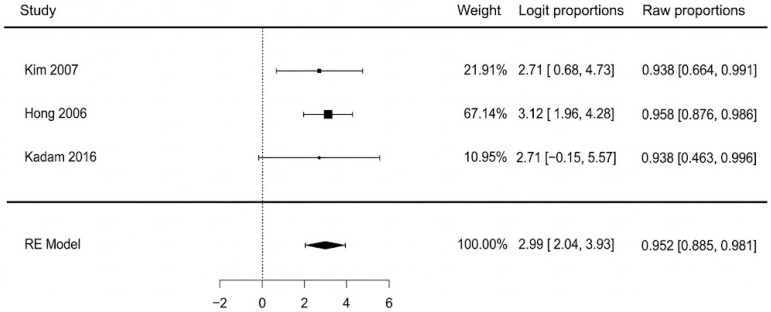
Return to ambulation [41,42,43].

The pooled analysis of three studies found that 95.2% of patients successfully returned to ambulation (95% CI: 88.5–98.1%). There was no heterogeneity observed among the studies (I^2^ = 0%, *p* = 0.923), indicating excellent consistency across the results. Visual inspection of the funnel plot and a non-significant Egger’s test (*p* = 0.712) revealed no evidence of publication bias. Pooled means are presented in Figure 8.

The pooled results for patient characteristics, defect and flap details, and additional clinical outcomes are summarized in Table 3.

The analysis of baseline patient data revealed a high pooled prevalence of critical comorbidities, including osteomyelitis (63.7%) and peripheral arterial disease (54.3%), though these findings had significant heterogeneity (I^2^ > 75%), indicating considerable variation in patient severity across the included cohorts. The mean glycated hemoglobin was 9.9%.

Regarding the defects, the most common locations for reconstruction were the dorsum of the foot (40.9%) and the plantar surface (38.6%). The mean flap area used was large, at 103 cm^2^, but this also varied substantially between studies (I^2^ = 97.6%).

Key clinical outcomes demonstrated low rates of postoperative complications. The pooled flap infection rate was 5.1% and the vascular insufficiency rate was 6.9%, with both outcomes showing low to no heterogeneity. The ulcer recurrence rate was also very low at 1.7%. The mortality rate, reported by a single study of 16 patients, was 6.2%. A significant functional outcome was the high rate of return to gait, which was 95.2% with no heterogeneity between the studies (I^2^ = 0%), indicating a highly consistent and positive result.

Only two of the included studies, Yang 2024 and Wen 2025 [44,45], reported on functional outcomes. Data collection was accomplished using two different scaling methods, namely the ASAMI (Association for the Study and Application of the Methods of Ilizarov) score and the AOFAS (American Orthopaedic Foot and Ankle Society) score. Due to differences in their reporting methods, the data could not be pooled and are presented here as a narrative summary.

The ASAMI functional score assesses a patient’s clinical recovery after limb reconstruction. It evaluates key practical outcomes, including pain, joint stiffness, the patient’s ability to resume daily activities and work, and cosmetic satisfaction. Based on these factors, the functional result is graded on a scale from excellent to poor, providing a standardized measure of the procedure’s success from the patient’s perspective [46]. Yang et al. [44] evaluated 31 diabetic foot patients 12 months after surgery and reported their ASAMI functional outcomes categorically. The results were presented in Table 4.

The American Orthopaedic Foot & Ankle Society (AOFAS) clinical rating system is a 100-point score widely used to evaluate functional outcomes of the foot and ankle. It integrates patient-reported assessments of pain and function with a clinician’s objective evaluation of alignment. Higher scores indicate better outcomes, making it a standard tool in research for quantifying patient recovery and comparing surgical results [47]. In the study by Wen et al. [45], 24 patients were evaluated using the AOFAS score preoperatively and again at a 6-month follow-up. The analysis revealed a substantial and statistically significant improvement in patient function. The mean score increased from a preoperative value of 63.5 (classified as a moderate result) to 90.8 (classified as an excellent result) at six months post-surgery.

Despite the differences in reporting, the findings from both studies consistently indicate positive functional and patient-reported outcomes following ALT flap reconstruction for diabetic foot defects. Collectively, this evidence suggests that patients undergoing ALT flap reconstruction can expect a high likelihood of achieving excellent functional status and a successful return to ambulation. The lack of standardized PROM reporting, however, highlights a gap in the literature and underscores the need for future studies to use consistent outcome measures.

The overall confidence in the evidence for the primary and secondary outcomes was evaluated using the GRADE system. All included studies were non-randomized retrospective cohorts, resulting in an initial certainty level of “Low” for each outcome. Upon examination, the confidence in all synthesis outcomes was downgraded to “Very Low” level of certainty, mostly due to significant risk bias from the included retrospective studies and considerable imprecision stemming from small sample sizes and large confidence intervals.

## 4. Discussion

This systematic review and single-arm meta-analysis synthesized data from six retrospective cohort studies, collectively including 162 patients who underwent anterolateral thigh free-flap reconstruction for diabetic foot defects. The analysis of the primary outcome, total flap failure, demonstrated favorable results, with a pooled rate of 5.2% (95% CI: 2.5–10.6%), a finding that was highly consistent across all studies (I^2^ = 0%). The pooled incidence of partial flap necrosis was 13.0% (95% CI: 6.3–25.1%), resulting in an overall weighted flap success rate of 81.8%. A meta-analysis reported by Reed et al., focusing on free-flap DFU reconstruction, shows a similar complete flap failure rate of 9.95% [24]. Moreover, Fitzgerald O’Connor et al. observed a total flap survival rate of 91.9% in a systematic review addressing free tissue transfer in diabetic lower extremity wounds, but with no specificity to the type of flap used [29]. Other studies examining pedicled flap outcomes in diabetic lower limb reconstruction showed complete necrosis rates of 3.5–3.7% and partial necrosis rates of 10.2–31.4%, comparable to our analysis’ results [48,49]. Thus, we can deduce that free ALT flaps represent a solid reconstruction option for diabetic foot ulcers.

Key secondary outcomes also yielded positive and consistent findings. Notably, the pooled rate of return to ambulation was 95.2% (95% CI: 88.5–98.1%), with no heterogeneity observed (I^2^ = 0%). This rate was observed to be significantly higher compared to other data reported in the literature. For example, just 78% of patients achieved full weight-bearing in a study by Hutting et al., while Kavarthapu reported a postoperative ambulation rate of only 71% [50,51].

Moreover, postoperative complications and long-term sequelae were infrequent. The pooled incidence of flap infection was low at 5.1%, as was flap compromise due to vascular insufficiency at 6.9%, with the latter showing no heterogeneity (I^2^ = 0%). Critically for this patient population, the pooled ulcer recurrence rate was exceptionally low at 1.7% (I^2^ = 0%), and the surgical revision rate was 7.7% (I^2^ = 0%), lower than a similar study by Burkhard et al. analyzing free flap outcomes in head and neck reconstruction with a revision rate of 32% [52].

These outcomes were achieved in a high-risk patient population, characterized by a high prevalence of deep infection, evidenced by a pooled osteomyelitis rate of 63.7%. Osteomyelitis complicating diabetic foot ulcers (DFUs) significantly increases the risk of amputation and mortality. Surgical debridement is mandatory in order to obtain successful infection clearance. Bone loss is often managed with antibiotic-loaded bone substitutes, such as gentamicin-loaded biocomposites or autografts if available. Regardless of the surgical approach, prolonged antimicrobial therapy (at least 6 weeks post-implant removal or 12 weeks with retention) is recommended [53]. Notably, significant statistical heterogeneity was observed for this parameter (I^2^ = 76.5%), indicating that the prevalence varies considerably across different patient populations. This variability is highlighted by the findings of Yan et al., who observed an osteomyelitis rate of 19.8% in patients with diabetic foot ulcers [54].

Furthermore, the cohort was characterized by significant macrovascular comorbidity, with a pooled peripheral arterial disease (PAD) prevalence of 54.3%. It is important to acknowledge that the high heterogeneity observed for PAD (I^2^ = 99.1%) indicates substantial variation in patient severity across the included studies. Other systemic risk factors were also prevalent, including chronic kidney failure (16.9%), high rates of tobacco consumption (41.1%), and poor glycemic control (pooled mean HbA1c 9.9%). Chronic kidney disease (CKD) is highly interconnected with the evolution and prognosis of diabetic foot ulcers. Up to 40% of people with diabetes develop CKD, and 19–34% experience a DFU in their lifetime [55]. CKD is a major independent risk factor for developing a DFU, with the risk progressively increasing as kidney function declines [56,57]. Even moderate CKD, defined as an eGFR below 60 mL/min/1.73 m^2^, significantly raises the risk of both DFU and lower-extremity amputation [58]. Furthermore, CKD is also a predictor of poor outcomes after interventions, such as revascularization, for diabetic foot disease [59].

The severity of the cases is also reflected in the defects themselves, which were extensive (mean flap area 103 cm^2^) and situated in challenging anatomical locations such as the plantar surface (38.6%) and the dorsum of the foot (40.9%). This is emphasized in comparison with other studies’ results, such as a paper by Rahma et al. reporting a mean ulcer size of just 0.55 cm^2^, while Huang et al. observed a mean ulcer area of 4.8 cm^2^ [60,61].

The functional success of ALT flaps in reconstructing diabetic foot defects is further supported by the narrative data from two studies regarding patient reported outcome measures: Yang et al. [44] reported that 21 of 31 diabetic patients (67.7%) achieved “Excellent” or “Good” ASAMI functional scores 12 months post-surgery, while Wen et al. [45] documented a significant mean improvement in AOFAS scores from 63.5 preoperatively to 90.8 at 6-month follow-up. This demonstrates that ALT flap reconstruction does not merely salvage the limb anatomically but also successfully restores function, which is a primary objective of treatment. This statement is further strengthened by studies such as one by Chang et al., demonstrating that free-flap reconstruction of diabetic feet significantly improves patient quality of life [62]. Collectively, these estimates position the ALT flap as a robust and effective primary tool for achieving both durable limb preservation and functional recovery in complex diabetic foot defects.

To our knowledge, this is the first systematic review and single-arm meta-analysis to quantitatively synthesize the outcomes of the anterolateral thigh free-flap specifically for diabetic foot reconstruction. A key strength of our analysis is the high consistency found for the primary outcome of total flap failure and the critical secondary outcome of return to ambulation, both of which demonstrated no statistical heterogeneity (I^2^ = 0%). This suggests that the positive performance of the ALT flap is a robust finding across different centers, even within the highly diverse patient populations included.

However, this review has several significant limitations. The primary issue is the poor methodological quality of the six included studies, all of which were retrospective cohorts with a high risk of selection and reporting bias. Additionally, the assessment of functional recovery was constrained by the lack of usage of standardized outcome measures; only two studies utilized validated scoring systems (ASAMI and AOFAS), limiting our ability to meaningfully measure patient-reported outcomes. Another critical limitation is the geographic restriction of the data, as all included studies originated from single centers in Asia. This introduces potential bias regarding patient characteristics which may differ from Western populations. Furthermore, many of these institutions are recognized as high-volume microsurgical hubs. Therefore, the reported outcomes may not be fully reproducible in lower-volume centers globally. Moreover, this study’s single-arm design lacks a control group, making it impossible to draw conclusions about comparative effectiveness. Finally, while there was high heterogenicity in baseline patient comorbidities, the outcomes themselves were compellingly consistent, suggesting the flap’s success is robust despite these variations.

Even though ALT free-flap has proven an excellent option in DFU reconstruction, the field requires higher-quality, prospective, and multicenter studies. Ideally, future research should also include comparative trials that weigh the ALT flap against other reconstructive techniques. Finally, for the benefit of future analyses and to better understand the patient experience, we strongly recommend the adoption of standardized functional and patient-reported outcome measures across all studies in this area.

## 5. Conclusions

In conclusion, the anterolateral thigh free-flap emerges from this analysis as a highly reliable and effective option for reconstructing complex diabetic foot wounds. The evidence synthesized here shows a very low risk of complete flap failure, indicating that the procedure is consistently successful in a technical sense. Moreover, this surgical success translates directly into outstanding functional recovery. This is a remarkable achievement, as the procedure was also associated with a low incidence of postoperative complications and, critically, a very low rate of ulcer recurrence over the long term.

## Figures and Tables

**Table 2:** Heatmap of Risk of Bias Assessment (green background—reported adequate, yellow background—reported inadequate, red background—not reported).

	Kim 2007 [42]	Hong 2006 [41]	Hong 2005 [40]	Kadam 2016 [43]	Yang 2024 [44]	Wen 2025 [45]
1. A clearly stated aim	2	2	2	1	1	2
2. Inclusion of consecutive patients	2	1	2	2	2	1
3. Prospective collection of data	0	0	0	0	0	0
4. Endpoints appropriate to the aim of the study	1	1	1	1	2	2
5. Unbiased assessment of the study endpoint	0	0	0	0	0	0
6. Follow-up period appropriate to the aim of the study	2	1	2	0	2	2
7. Loss to follow-up less than 5%	0	2	2	2	2	2
8. Prospective calculation of the study size	0	0	0	0	0	0
Item 9–12 only for comparative studies						
9. An adequate control group					1	1
10. Contemporary groups					2	2
11. Baseline equivalence of groups					2	2
12. Adequate statistical analyses					2	2
TOTAL MINORS score	7	7	9	6	16	16
Maximum possible score	16	16	16	16	24	24

**Table 3:** Summarization of results.

Outcome	No. of Studies (k)	Total Patients (N)	Pooled Estimate (95% CI)	Heterogeneity (I^2^)
Primary outcomes				
Complete flap necrosis	6	162	5.2% [2.5%, 10.6%]	0%
Partial flap necrosis	4	131	13% [6.3%, 24.8%]	46.8%
Patient characteristics				
Age (years)	5	91	54.9 [49.7, 60.2]	90.1%
Prevalence of osteomyelitis	4	104	63.7% [31.4%, 87%]	76.5%
Glycated Hemoglobin (%)	2	40	9.9% [8.5%, 11.5%]	75.3%
Peripheral arterial disease	2	50	54.3% [−35%, 144%]	99.1%
Chronic kidney failure	2	41	16.9% [5.5%, 28.2%]	0%
Chronic heart failure	1	34	14.7%	N/A
Tobacco consumption	1	34	41.1%	N/A
Defect location				
Foot plantar surface	4	81	38.6% [28.6%, 49.8%]	0%
Dorsum of foot	3	74	40.9% [20.8%, 64.5%]	71.99%
Heel	3	57	27% [8%, 61.2%]	75.79%
Ankle	3	74	15.6% [4.3%, 42.9%]	70.11%
Calf	2	50	3.7% [−4%, 11.6%]	0%
Flap characteristics				
Area (cm^2^)	5	138	103 [50, 155]	97.6%
End-to-end anastomosis	2	23	11.4% [−16%, 38.8%]	68.7%
End-to-side anastomosis	2	23	88.6% [61.2%, 116%]	68.7%
Anterior tibial artery as receiving vessel	3	57	42.2% [24.4%, 62.3%]	45.2%
Posterior tibial artery as receiving vessel	3	57	55.6% [30.4%, 78.3%]	65.17%
Peroneal artery as receiving vessel	1	16	0%	N/A
Mean pedicle length (cm)	1	16	14.1	N/A
Number of perforators/flaps	1	16	1.125	N/A
Outcomes				
Flap infection	4	145	5.1% [1.6%, 14.7%]	40.2%
Vascular insufficiency	3	74	6.9% [2.9%, 15.4%]	0%
Ulcer recurrence	2	78	1.7% [−2.6%, 6.1%]	0%
Revision rate	2	87	7.7% [1.5%, 13.9%]	0%
Wound healing success rate	1	24	100%	N/A
Time to complete wound healing (weeks)	2	58	7.2 [2.3, 12.1]	95.1%
Mean follow-up (weeks)	2	44	119 [114, 125]	0%
Mean length of stay (weeks)	1	71	3.5	N/A
Gait return	3	94	95.2% [88.5%, 98.1%]	0%
Mortality	1	16	6.20%	N/A

**Table 4:** ASAMI functional results.

ASAMI Functional Results	Number of Patients
Excellent	8
Good	13
Fair	7
Poor	3

## Data Availability

The raw data supporting the conclusions of this article will be made available by the authors on request.

## Supplementary Materials

The following supporting information can be downloaded at: https://www.mdpi.com/article/10.3390/jcm14238481/s1. PRISMA 2020 Checklist [36].

## Abbreviations

The following abbreviations are used in this manuscript:

AOFAS	American Orthopaedic Foot and Ankle Society
ALT	Anterolateral thigh
ASAMI	Association for the Study and Application of the Methods of Ilizarov
CKD	Chronic kidney disease
DFU	Diabetic foot ulcer
eGFR	Estimated glomerular filtration rate
HbA1c	Glycated hemoglobin
MeSH	Medical Subject Headings
MINORS	Methodological Index for Non-Randomized Studies
MMP	Matrix metalloproteinase
PAD	Peripheral arterial disease
PRISMA	Preferred Reporting Items for Systematic Reviews and Meta-Analyses
PROM	Patient-reported outcome measure
RCT	Randomized controlled trial
RE	Random-effect

## Appendix A

Full search strategies for databases used:

1. Embase

(‘diabetic foot’/exp OR (diabetic foot OR diabetic feet OR foot ulcer* OR diabetic ulcer* OR Charcot foot).ti,ab,kw.) AND (‘free tissue flap’/exp OR (free flap* OR free tissue transfer* OR microsurg*).ti,ab,kw.) AND (anterolateral thigh OR ALT OR ALT flap*).ti,ab,kw. AND (foot OR feet OR pedal OR plantar OR ankle).ti,ab,kw.

2. Scopus

TITLE-ABS-KEY(

(“diabetic foot” OR “foot ulcer*” OR “diabetic ulcer*” OR “charcot foot”)

AND

(“free flap*” OR “free tissue transfer*” OR microsurg*)

AND

(“anterolateral thigh” OR “ALT” OR “ALT flap*”)

AND

(foot OR feet OR pedal OR plantar OR ankle)

)

3. Cochrane Library

(“Diabetic Foot” OR “diabetic foot” OR “diabetic feet” OR “foot ulcer*” OR “diabetic ulcer*” OR “Charcot foot”) AND (“Free Tissue Flaps” OR “free flap*” OR “free tissue transfer*” OR “microsurg*”) AND (“Anterolateral Thigh” OR “anterolateral thigh” OR “ALT” OR “ALT flap*”) AND (“foot” OR “feet” OR “pedal” OR “plantar” OR “ankle”)

4. Web of Science Core Collection

TS=(

(“diabetic foot” OR “foot ulcer*” OR “diabetic ulcer*” OR “charcot foot”)

AND

(“free flap*” OR “free tissue transfer*” OR microsurg*)

AND

((“anterolateral” NEAR/3 “thigh”) OR “ALT” OR “ALT flap*”)

AND

(foot OR feet OR pedal OR plantar OR ankle)

)
